# Combining multi-mutant and modular thermodynamic cycles to measure energetic coupling networks in enzyme catalysis

**DOI:** 10.1063/1.4974218

**Published:** 2017-01-26

**Authors:** Charles W. Carter, Srinivas Niranj Chandrasekaran, Violetta Weinreb, Li Li, Tishan Williams

**Affiliations:** Department of Biochemistry and Biophysics, University of North Carolina at Chapel Hill, Chapel Hill, North Carolina 27599-7260, USA

## Abstract

We measured and cross-validated the energetics of networks in *Bacillus stearothermophilus* Tryptophanyl-tRNA synthetase (TrpRS) using both *multi-mutant* and *modular* thermodynamic cycles. Multi-dimensional combinatorial mutagenesis showed that four side chains from this “molecular switch” move coordinately with the active-site Mg^2+^ ion as the active site preorganizes to stabilize the transition state for amino acid activation. A *modular* thermodynamic cycle consisting of full-length TrpRS, its Urzyme, and the Urzyme plus each of the two domains deleted in the Urzyme gives similar energetics. These dynamic linkages, although unlikely to stabilize the transition-state directly, consign the active-site preorganization to domain motion, assuring coupled vectorial behavior.

## INTRODUCTION

One of our oldest and most inclusive insights about proteins is that protein folding coordinates the behavior of the amino acids linked together in the polypeptide chain. Proposals implicating the resulting cooperative protein motions, or “dynamics,” in enzyme catalysis are commonplace.^1–11^ A central notion underlying such proposals is that low frequency motions—loop closing, domain motion, etc.—transmit the kinetic energy along directions aligned with chemical changes, thereby contributing to the overall rate acceleration.

Such proposals have been challenged, however, for three main reasons.^12^ (i) Frequencies of domain motions are many orders of magnitude slower than those of the chemical reactions. (ii) Forces that can be imposed with such motions are substantially weaker than those necessary to stabilize the transition states. (iii) In order to definitively implicate motions in catalysis, it is necessary to map the accurate free energy landscapes of chemical reactions and how they integrate with the protein-dynamical changes, and cases where these have been done do not reveal a significant catalytic contribution.

Nonetheless, many enzymes, especially NTP (nucleotide triphosphate)-dependent motors, synthetases, polymerases, and signaling GTPases, exhibit multi-state, *vectorial* behavior^13–19^ in which biological function depends critically on coupling catalysis to conformational changes. Purposes served by such coupling include many of the most interesting aspects of biology. For this reason, the creation of, and transitions between, these multiple states are biologically relevant. It is therefore important to determine the mechanisms by which conformational transitions can be coupled to active-site chemistry.^13,14,20^

Our work on *Bacillus stearothermophilus* tryptophanyl-tRNA synthetase (TrpRS) occupies a specialized niche amidst these questions. We try to answer such questions as:

(i) What remote parts of a protein exert a substantive influence on catalysis?
(ii) What techniques are necessary to verify these effects?
(iii) What is the magnitude of these influences?
(iv) How cooperative are they?
(v) What is their functional significance?

TrpRS furnishes an attractive laboratory model for exploring long-range coupling comprehensively.

Our interest in these questions was initially stimulated by communications with Arieh Warshel, who has argued forcefully that the essence of catalysis is electrostatic stabilization of the transition state^21–23^ and cannot arise from dynamical effects. Reviewing an earlier paper of ours,^24^ he challenged our contention that long-range forces might be involved in catalysis, and argued that the TrpRS rate acceleration was due primarily to electrostatic stabilization by the single Mg^2+^ ion.

Testing that idea experimentally, we found that only about a third of the overall transition-state stabilization could be attributed to the Mg^2+^ ion.^25^ This posed two questions:

(i) What accounts for the metal-independent two-thirds of the transition-state stabilization?
(ii) How does metal-protein coupling work if Mg^2+^ does not interact with the protein, has very little effect on the rate in water, but accelerates the rate 10^5^-fold in the presence of TrpRS?

The first question remains to be answered, although we have taken some steps^26,27^ in that direction. We tentatively answer the second question here. We conclude that Warshel is correct that domain movements themselves cannot directly stabilize transition states. However, what he calls the “active site pre-organization”^28^ appears to occur only during the domain movement, which has the effect of synchronizing catalysis with domain movement. The resulting conditional dependence of catalysis is what couples catalysis to domain movements,^20,29,30^ enabling the vectorial behavior. Readers may wish to refer to Figs. 10 and 11, which are discussed in detail later on, and provide a useful visualization of the proposed reaction path.

## RESULTS

TrpRS uses Adenosine 5′ triphosphate (ATP) to activate the carboxyl group of the amino acid tryptophan to form Trp-5′ adenylate plus pyrophosphate (PPi). This reaction is the *sine qua non* of protein synthesis, and therefore of fundamental biological significance. Domain movements along the TrpRS structural reaction profile were elucidated by solving the crystal structures of TrpRS without ligands^31^ and complexed to substrates—tryptophan, ATP;^32^ non-reactive substrate analog combinations—tryptophanamide plus ATP;^32^ tryptophan plus Adenosine-5′ monophosphate (AMP) plus PPi;^33^ a transition-state analog—adenosine-5′tetraphosphate;^34^ and products—trp-5′AMP,^35,36^ trp-5′sulfoamyl-AMP, trp-methenyl-AMP;^37^ representing different states along the reaction path. These crystal structures group into three distinct, narrowly defined conformations that correspond to nodes connected by the three canonical stages of enzyme catalysis: induced-fit active-site assembly, catalysis, and product release.^38^ In this respect, the three resulting “states”—open, pre-transition state (PreTS), and Products—correspond functionally and structurally to the three conformations observed in the successive stages of the chemical reactions of the ATP by the catalytic F1-ATPase β-subunit.^39^ Specifically, an open conformation is associated with the unliganded state, a closed, twisted conformation is associated with the ATP bound state, and a closed, untwisted conformation is associated with the aminoacyl-AMP-bound state. We previously commented on the possible relationship between the three-state behavior and free energy transduction.^38^

The three TrpRS conformations are produced by the relative rigid-body movement of the anticodon-binding domain (ABD) relative to the catalytic domain.^33^ The induced-fit and catalytic transitions are rate-limited by high-energy conformational transition states^24,40^ along the conformational free energy surface. We identified the structures responsible for these high-energy conformations using searches for the most probable paths that connect the respective ground-state conformations.^30,41^ An important barrier to domain movement associated with induced-fit is a change in aromatic side chain packing within the D1 switch, a motif located in the N-terminal crossover connection of the Rossmann fold.^41,42^

**FIG. 1.**
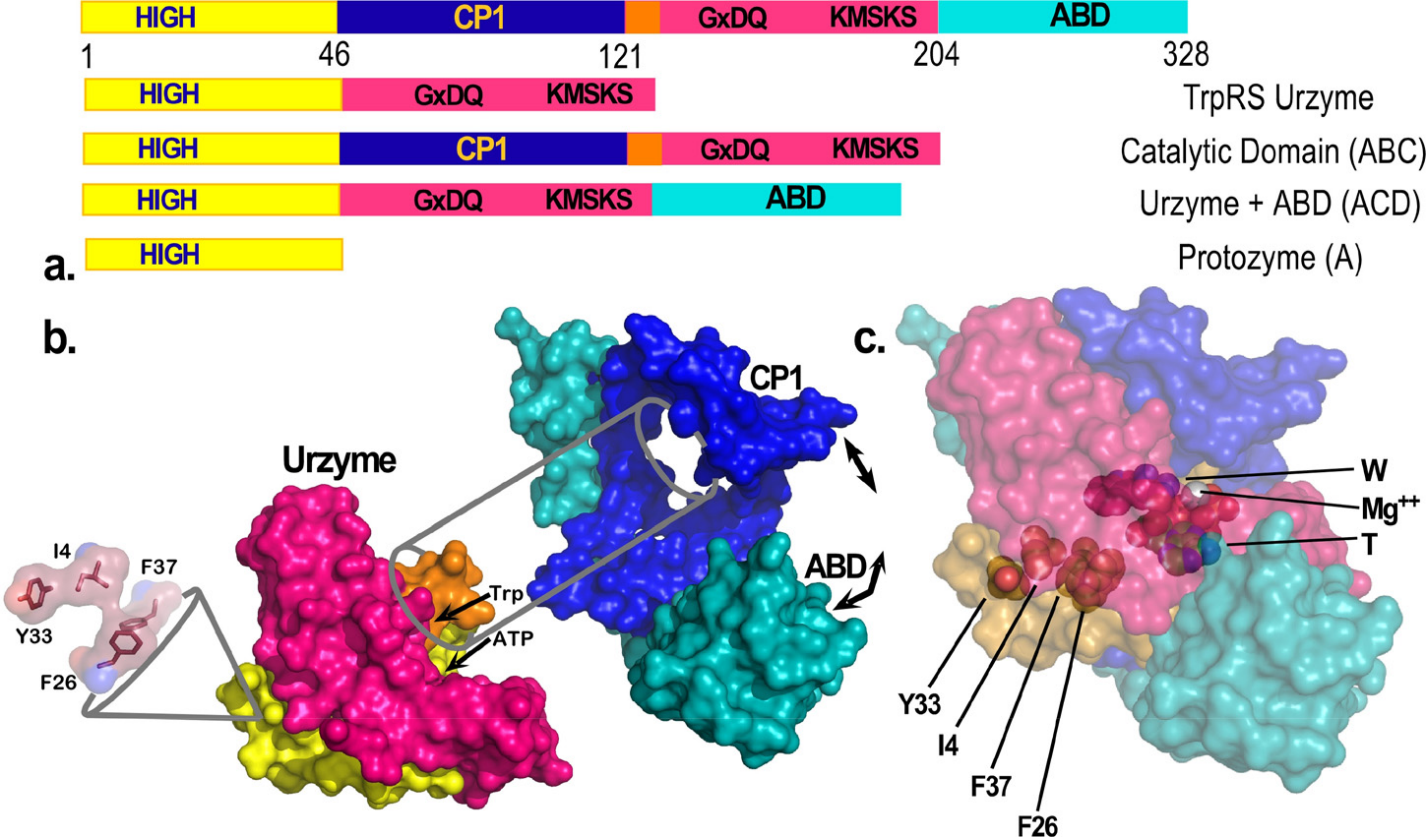
TrpRS hierarchy of functional modules. (a) Schematic of sequential order of TrpRS modules: Protozyme (yellow), Urzyme, (yellow plus magenta), CP1 (dark blue), and anticodon-binding domain (teal). (b) Fitting of the D1 Switch into the Urzyme, and of the Urzyme into the framework imposed by CP1 (blue) and the ABD domain (teal). The D1 Switch forms the helix-strand corner of the first crossover connection (yellow). The second crossover connection of the Rossmann fold (red) follows the CP1 insertion and is part of the Urzyme. Correlated motions of CP1 and the ABD are indicated by arrows. (c) Relationship between the D1 Switch residues and the active-site. W is tryptophan, T is ATP.

Seeking to understand the evolutionary sources of TrpRS catalysis, we have experimentally deconstructed the monomer (Fig. 1) into three nested, catalytically active components—Protozyme (46 residues), Urzyme (130 residues), and Catalytic Domain (204 residues)—which, together with the anticodon-binding domain (ABD) form the full-length TrpRS monomer.^26^ The Protozyme and Urzyme retain a considerable catalytic activity and hence likely represent ancestral forms of contemporary aminoacyl-tRNA synthetases. Full-length synthetases combine the Urzyme with two additional modules added later in evolution: connecting peptide 1 (CP1), which completes the catalytic domain and provides the context for editing domains in the larger synthetases specific for aliphatic side chains; and the anticodon-binding domain, which enables recognition of the tRNA anticodon (Fig. 1(a)). The CP1 module does not actually move as a rigid body with the Urzyme, but moves relative to it. We, and others, have used computational methods to identify the correlated motions of the ABD domain and CP1 in several of the Class I synthetases.^40,43^

CP1 and the ABD together form a “cone” that is fitted around the Urzyme (Fig. 1(b)). The active-site opening is accessible only through this cone, which therefore appears to physically constrain the relative positions of the Urzyme's modular ATP and amino acid binding sites. The active site is thus sensitive to the configuration of the CP1 and ABD modules in full-length TrpRS. We have measured the impact of these interactions on catalysis^20,25,29^ and specificity.^30,44^

### Thermodynamic cycles measure the coupling energies

**FIG. 2.**
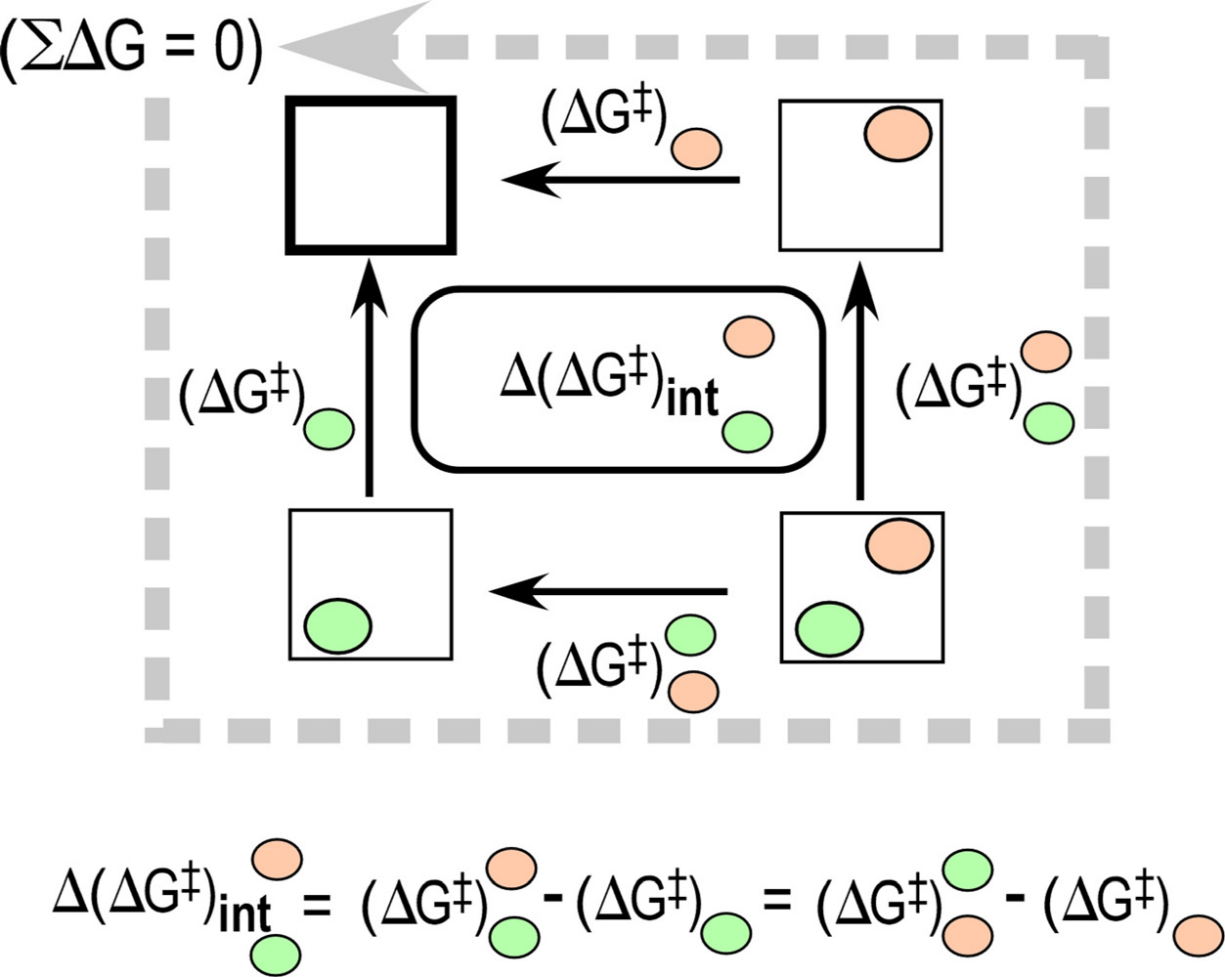
Schematic of a double mutant thermodynamic cycle. The factorial design of this experiment emphasizes measurement of the impact of mutation at one site (green ellipse) in the context of mutation at a second site (salmon ellipse). Rate changes between nodes along the edges are converted to free energies, ΔG^‡ ^= -RTln(k) = −0.592*ln(k). As the total free energy change is zero for the circuit, non-additivities of opposite vertical and horizontal edges are necessarily equal and furnish estimates of the coupling energies between the two perturbed sites.

A key concept underlying this work is that of “energetic coupling.” Coupling implies the existence of sources of free energy that enforce joint behaviors; such joint behaviors enable new, higher levels of functionality. It is generally true that coupling energies across a very broad spectrum of circumstances can be assessed from the non-additivity of expected contributions. A useful example is that the intrinsic proton angular momentum, or spin, cannot be explained using only the intrinsic properties of its three constituent quarks, but instead requires an energetic coupling between them and the gluons that hold them together.^45^ In nuclear physics, the data come from an inelastic electron and proton scattering experiments.^46^ In structural biology, non-additivities are evaluated quantitatively for combinatorial perturbations using a device that in a biophysical context is called a thermodynamic cycle (Fig. 2).

A thermodynamic cycle is a 2^N^ factorial experiment that tests all possible combinations of specific perturbations for their impact on kinetic or thermodynamic behavior. Perturbations that have non-additive effects in contexts of other perturbations imply coupled interactions. Use of multi-mutant cycles was introduced in biochemistry by Jencks^47,48^ and exploited by Fersht soon after the development of directed mutagenesis enhanced their feasibility.^49–51^ High-order thermodynamic cycles have demonstrated three-dimensional coupling interactions capable of changing the direction of conformational equilibria by up to 10^5^-fold.^52^

The ^32^PPi exchange reaction used to assay the aminoacyl-tRNA synthetases is done under equilibrium conditions, so both K_M_ and k_cat_ are related to the thermodynamic values, ΔGK_M_ and ΔGk_cat_. Free energies are additive, so linear regression methods can solve the simultaneous equations necessary to estimate and assess the statistical significance of coefficients for main and higher-order interaction effects.^20^ The latter represent the accessible coupling energies for a given experimental design. Experimental perturbations according to a full-factorial design can thus be used to estimate both the strength and significance of energetic coupling between parts of a protein during enzyme-catalyzed reactions.

In the course of this work, we were led to investigate a series of apparently unrelated combinatorial perturbations. To our surprise, three qualitatively quite different thermodynamic cycles give the same quantitative energetic coupling during TrpRS catalysis.

### The TrpRS/Mg^2+^ cycle; Mg^2+^ ion accelerates amino acid activation ∼10^5^-fold in the TrpRS-catalyzed reaction, but only 5-fold in water

**FIG. 3.**
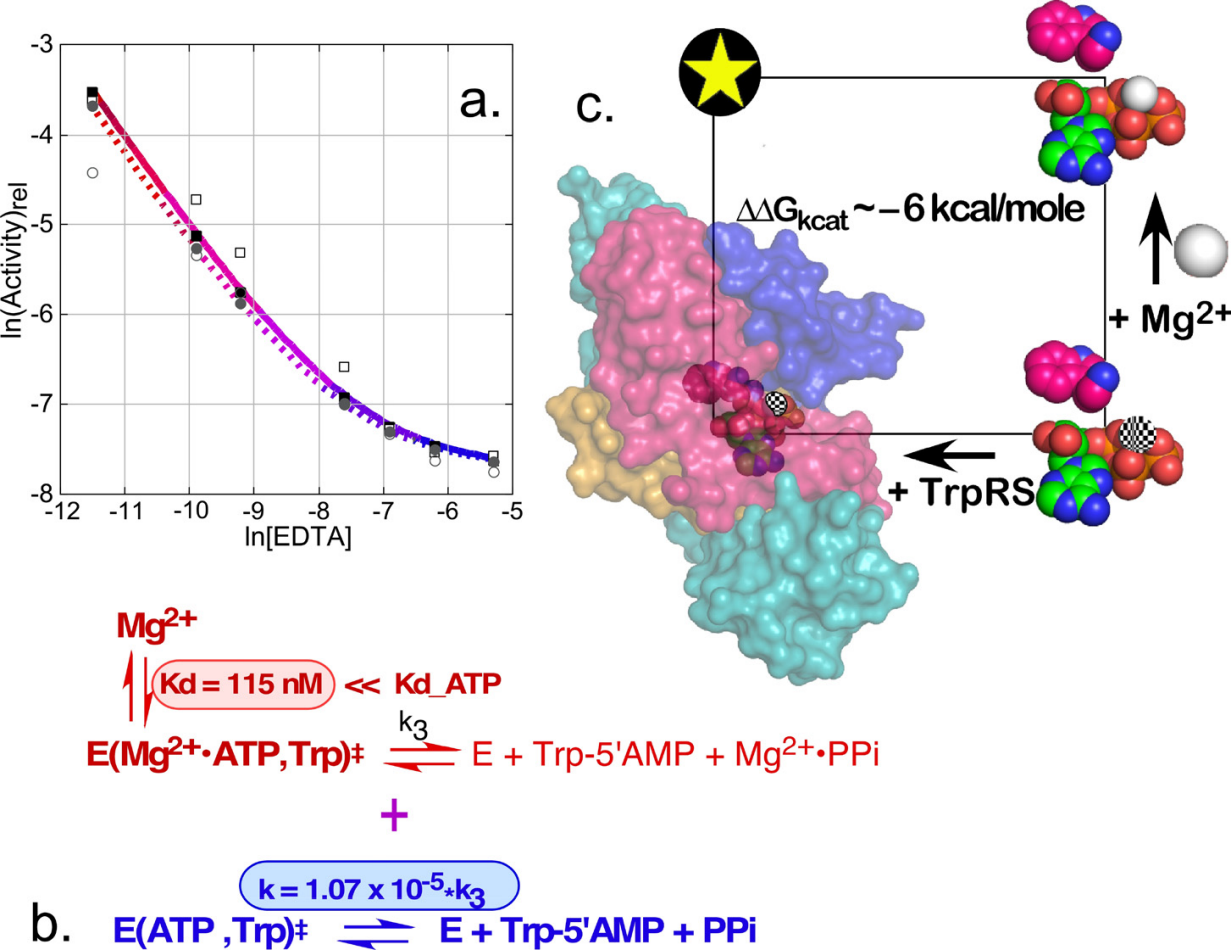
The TrpRS·Mg^2+^ cycle. (a) Experimental data supporting the conclusion that TrpRS induces a limiting rate acceleration in the absence of Mg^2+^, achieved by adding increasing amounts of EDTA to remove residual metal. Red coloring of the decay curve indicates residual catalytic assist by Mg^2+^ ion; Blue coloring indicates a plateau of catalysis by metal-free TrpRS. (b) Nonlinear fitting of the [EDTA]-dependence of the logarithm of TrpRS activity yields two parameters, K_D_^‡^, the dissociation constant for Mg^2+^ ion from the transition state (highlighted in red) and the maximum rate acceleration given by saturating Mg^2+^ concentrations (highlighted in blue) (adapted with permission from Weinreb and Carter, Jr., J. Am, Chem. Soc. **130**, 1488–1494 (2008). Copyright 2008 American Chemical Society). (c) Tryptophan activation is accelerated to very different extents by the addition of either TrpRS (10^9^-fold) or Mg^2+^ (5-fold). The two together induce an overall acceleration of 10^14^-fold. The ΔG_int_ = 6.4 kcal/mole. R^2 ^= 0.999 for the model, with P(F) = 0.0006.

Prompted by the suggestion that the TrpRS catalysis should be essentially eliminated in the absence of Mg^2+^ ion, we prepared extensively depleted stocks of enzyme, assay buffer, and reagents.^25^ The resulting assays exhibited reduced, but still substantial rate accelerations over the uncatalyzed rate, which we initially attributed to residual remaining Mg^2+^ ion (Fig. 3(b)). Titration of the assays with increasing amounts of ethylene diamine tetraacetate (EDTA) caused an additional rate reduction, but that reached a plateau beyond which there was, surprisingly, no further decrease.^25^

Iterated solution of the simultaneous binding equilibria using known dissociation constants for the Mg^2+^·ATP and Mg^2+^·EDTA equilibria allowed us to fit the activity data at two TrpRS concentrations to a nonlinear model with two parameters: the proportional decrease in rate in the absence, relative to that in the presence of Mg^2+^ ion (1.1 × 10^−5^) and the dissociation constant of Mg^2+^ from the transition-state complex (120 nM)^25^ (Fig. 3(c)). The former result implies that the metal accounts for only about 33% of the transition-state stabilization free energy, and is unexpected for several reasons: (i) It implies that the TrpRS active site alone is capable of ∼−12 kcal/mole of transition-state stabilization free energy, even in the absence of the metal; (ii) Mg^2+^ ion accelerates ATP-dependent reactions in water by only ∼5-fold.^53^ The two-factor thermodynamic cycle mapping the effects of TrpRS and Mg^2+^ ion thus exhibits a non-additivity of −6.4 kcal/mole, implying that the metal and protein are coupled by that energy in the transition state for ATP-dependent tryptophan activation.^25^

**FIG. 4.**
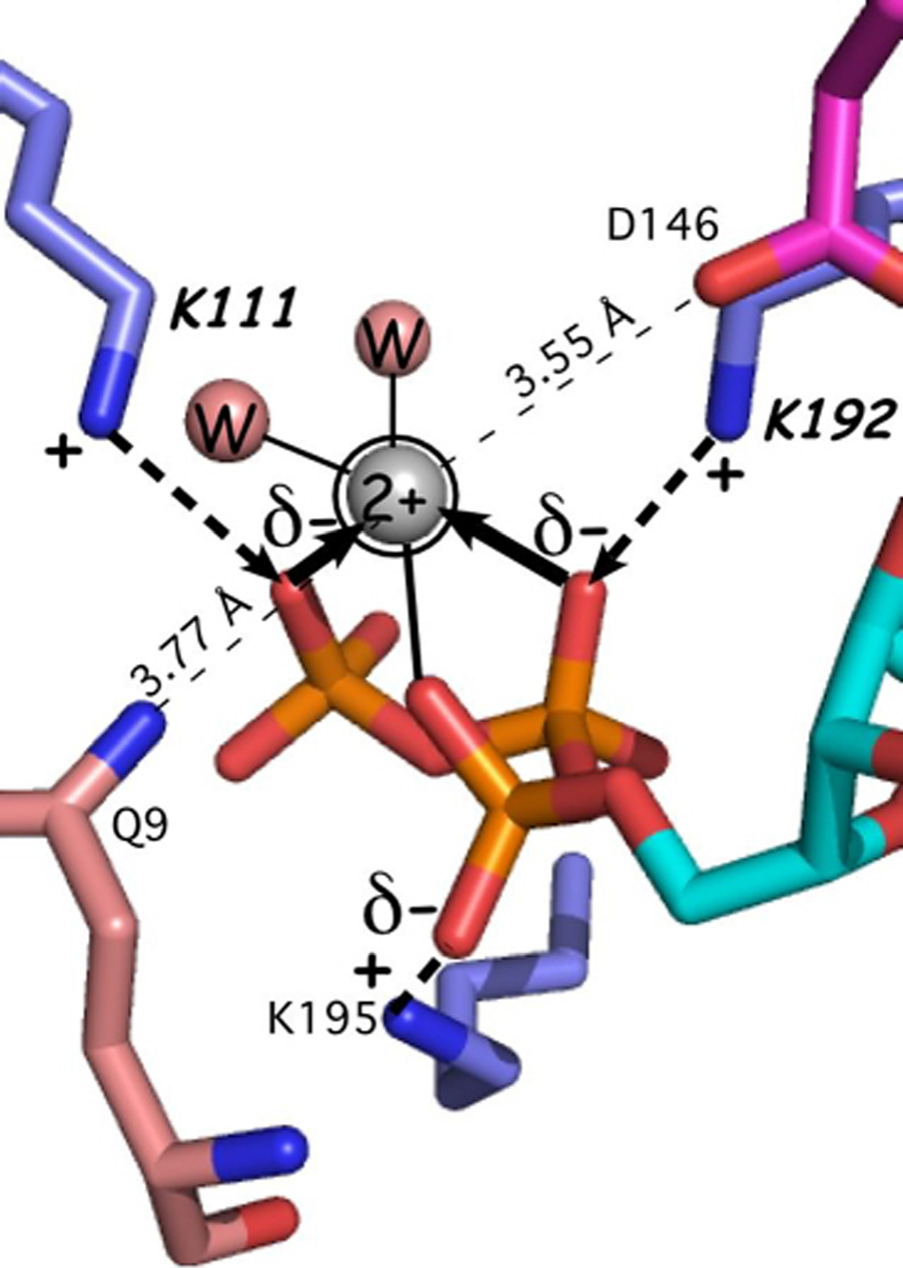
Coordination of the active-site Mg^2+^ involves no protein residues. The metal ion is seen only in TrpRS PreTS crystal structures,^32^ where it assumes an evidently strained configuration in which distances from each of three phosphate oxygen atoms are longer than those in Mg^2+^·ATP.^85^ The competition between Mg^2+^ and the lysine residues also weakens TrpRS affinity for ATP.^24^

Mg^2+^ has been observed in crystal structures only of the PreTS state. In those structures (1MAU, 1M83), it interacts directly only with three phosphate oxygen atoms and two water molecules, and not with any TrpRS functional groups. The absence of protein-metal contacts necessarily implied that the energy coupling them together arises from as yet unidentified sources that, in turn, interact with the Mg^2+^ ion indirectly, via the ATP phosphate oxygens. We first sought to identify these unknown coupling interactions within the active site, via combinatorial mutagenesis of the active-site lysine residues that are physically coupled to the metal because they compete with the metal for electrostatic interactions with phosphate oxygen atoms (Fig. 4).^29^ Those experiments showed that the Mg^2+^·protein interactions within the active-site actually retard the reaction,^29^ forcing us to look further afield for the sources of catalytic assist by Mg^2+^.

### Identification of the D1 switch

**FIG. 5.**
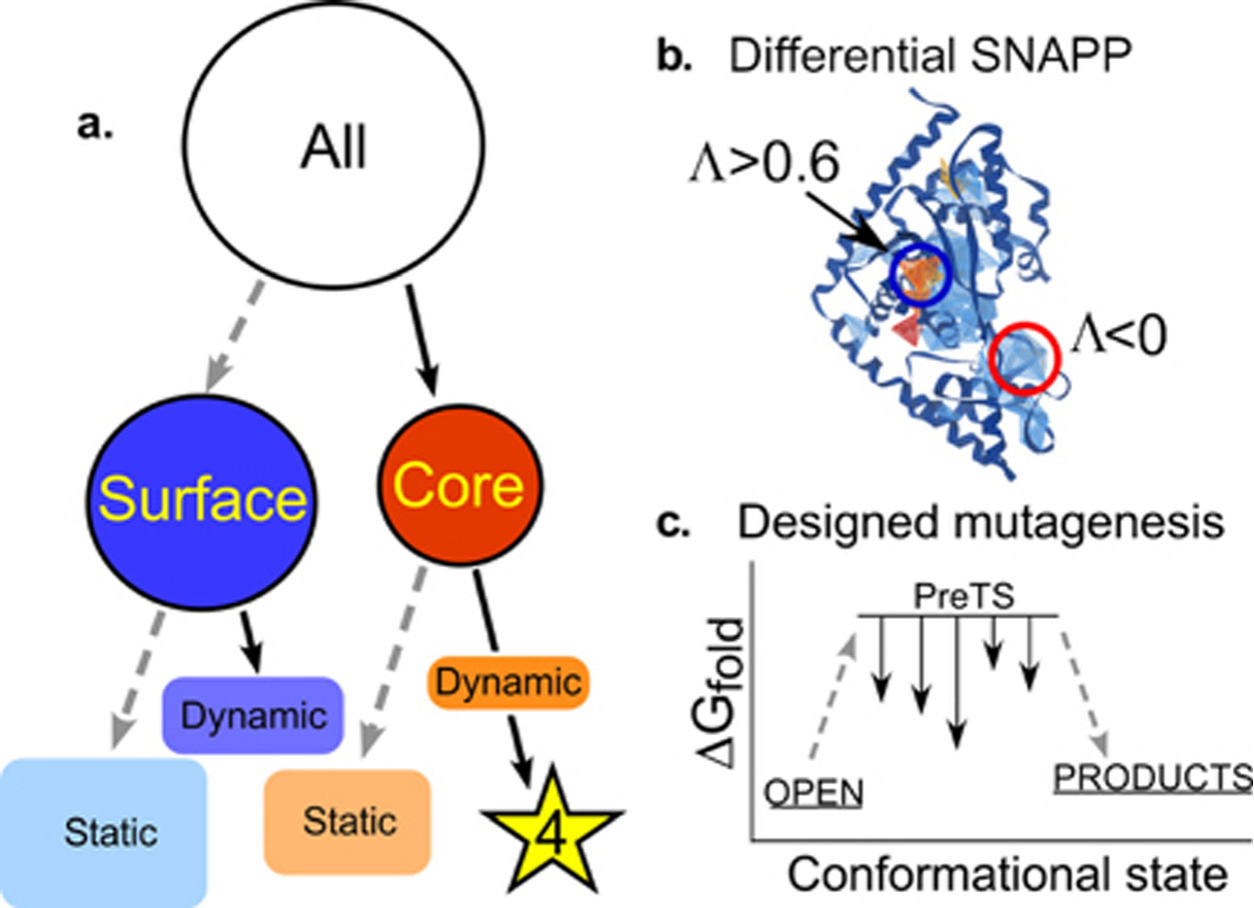
Identification of dynamic contacts with a two-stage bioinformatics filter. (a) Schematic of sequential identification of dynamic and static, core and surface interactions resulting in the identification of dynamic core residues. (b) Differential Delaunay tessellation and likelihood scoring identify simplices that rearrange partners in different conformations. Of the 250 simplices in the TrpRS monomer, 70 have high SNAPP log-likelihoods (Λ > 0.6; (red)) and 150 have negative log-likelihoods (Λ < 0.0; (blue)). The majority of those residues identified by SNAPP analysis are embedded within rigid bodies and these do not rearrange during catalysis. Of those “dynamic” simplices that do rearrange, 4 and 28, respectively, of each type, fall within the D1 Switch and also have mutations suggested by Rosetta in (c). (c) Protein multi-state design^59^ is used to identify the side chains whose mutation should stabilize the PreTS conformation, relative to the OPEN and PRODUCTS ground-states. Roughly 65% of the mutations suggested by Rosetta occur either in high SNAPP log-likelihood core tetrahedra (blue) or in negative log-likelihood tetrahedra, which are found at the protein surface (red).

To locate these distant interactions, we used a combination of two bioinformatic filters—one geometric, the other energetic—to identify side chains likely to influence conformational equilibria (Fig. 5).^24^ First, we quantitatively identified the local packing interactions that were invariant throughout the conformational cycle using Delaunay tessellation.^54–56^ Eliminating from consideration all invariant tetrahedra of nearest neighbors—those within rigid bodies—greatly reduced the number of such simplices that are “dynamic” and change during catalysis. Then, we used Rosetta^57–59^ to identify point mutants that might stabilize the high-energy PreTS state, relative to the OPEN and PRODUCTS ground states. The intersection of Venn diagrams identified by both Delaunay tessellation and by the multi-state design algorithm was surprisingly small,^24^ consisting of one large, centrally located dynamic packing motif and three smaller peripheral motifs. We called these dynamic motifs “Switches” D1–D4, from “Delaunay.”

These bioinformatic filters are discussed further in the Methods section. Their combined effects reduced the number of candidate residues to a small enough number that experimental perturbation was feasible. The majority of the residues that survived this two-stage filter were all part of the D1 switch, a packing motif at the C-terminus of the first alpha helix of the Rossmann fold. We subsequently identified this packing motif as one of the most widely distributed motifs in the proteome.^42^ The widespread phylogenetic distribution and key location of this motif in the first crossover connection of the Rossmann fold reinforced our surmise that the residues engaged in dynamic switching in this region might be responsible for the long-range coupling to the active-site Mg^2+^ ion. Although the D1 Switch motif consists of seven residues, we decided to mutate only four, because we sought a full factorial design, and 15 variants was an accessible number to construct and assay. Moreover, because the Rosetta multi-state design algorithm had given us the specific mutations for each site—I4V, F26L, Y33F, F37I—and because these mutations all consistently reduced differences between the Rosetta scores of the PreTS and Product structures, we used these specific residues, rather than alanine mutations.

### Combinatorial mutagenesis of D1 switch residues; long-range coupling to the catalytic Mg^2+^ ion

Alan Fersht used the term “multi-mutant cycle analysis” to describe the factorial directed point mutagenesis designs.^51^ Such experiments measure the coupling energy between individual residues and/or active site ligands. Extensive combinatorial multi-mutant cycle analysis of active-site functional groups subsequently showed that none of these coupling energies between protein and metal resides in the active-site residues,^29^ creating a strong argument for functionally relevant effects arising from parts of the protein distant from the active-site.

In order to detect coupling to the active-site metal, we first verified that the catalytic assist by Mn^2+^ ion was a suitable perturbation by showing that the metal substitution reduced activity to ∼15% of that with Mg^2+^ and that the concentration dependencies of catalysis by the two metals were very similar over a 10^−5^-fold range of metal concentrations, both exhibiting a maximal effect at approximately the same concentrations.^29^ All possible combinations of the four D1 mutations were constructed, purified, and their active fractions were determined by active-site titration.^60^

**FIG. 6.**
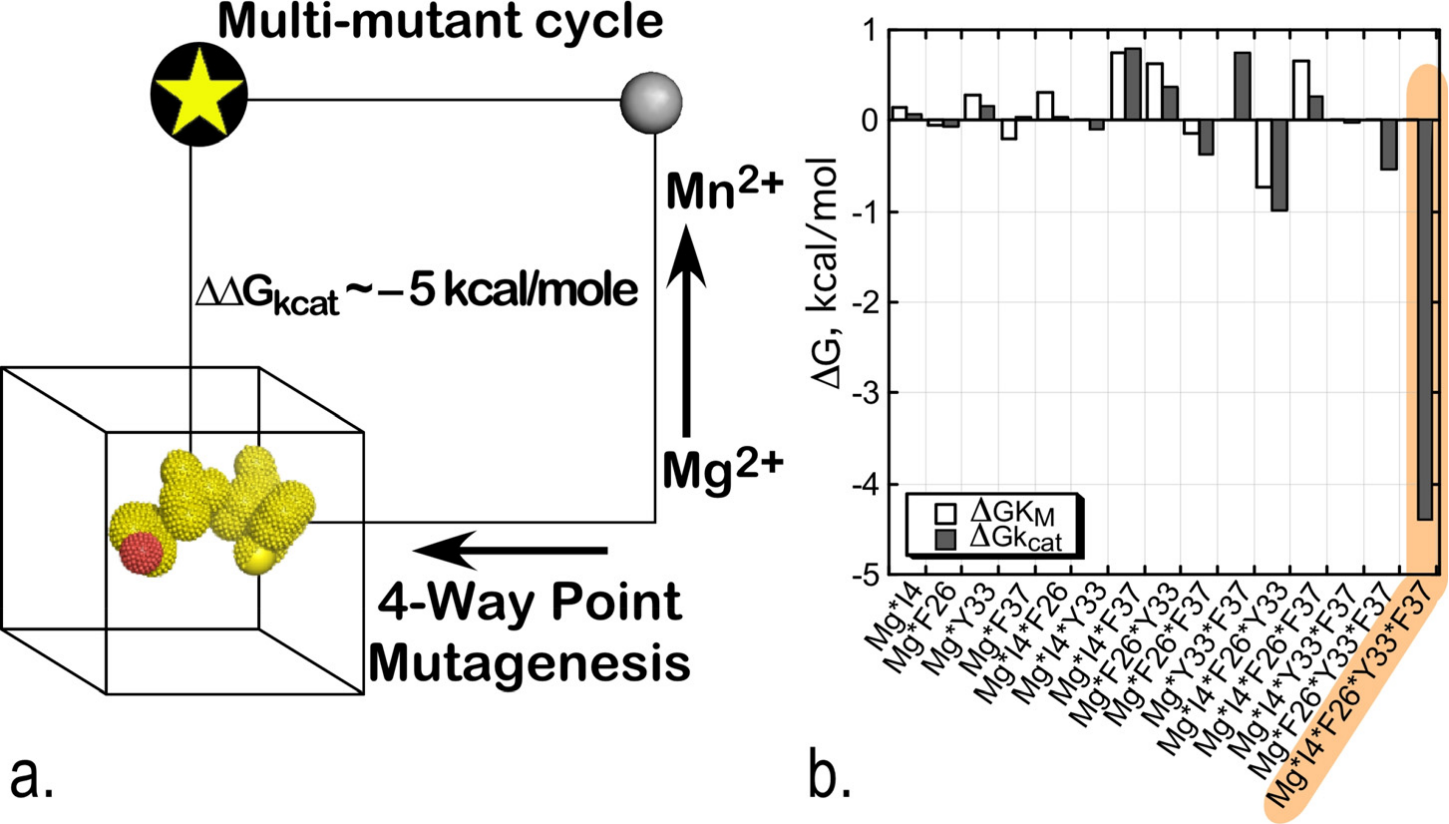
Combinatorial mutagenesis of four D1 Switch residues identify a strong, 5-way interaction (shaded amber) implying that all four mutated residues move coordinately with the active-site Mg^2+^ ion in the transition state for tryptophan activation.^20^ (a) Schematic of the 5-way thermodynamic cycle. (b) The combinatorial mutagenesis of the D1 residues, I4, F26, Y33, and F37, together with the substitution of Mg^2+^ with Mn^2+^ reveals that all five moieties are moving coordinately as the chemical transition state is stabilized. R^2 ^= 0.95 for the model, with P(F) < 0.0001, and Student t-test probabilities range between <0.001 to 0.05 for coefficients >0.15 kcal/mole.

The resulting experimental design involved 32 separate combinations of point mutant and metal (Mg^2+^ vs. Mn^2+^) and was thus perhaps the most ambitious of its kind yet attempted.^19,61^ All variants were assayed by ATP-dependent Michaelis-Menten steady-state kinetic analysis. Approximately, three-fold replication of the experimental data afforded a fit in which essentially all the main and higher-order interaction free energies of transition-state stabilization could be estimated with high statistical significance.^20^ Overall, the regression model for ΔGk_cat_ has coefficients with only modest magnitudes, of both signs, for the main effects and intermediate-order interactions. The 5-way interaction between the four permuted sites and the metal ion, however, contributes ∼−5 kcal/mole. The 5-dimensional multi-mutant cycle showed that ∼85% of the catalytic effect of Mg^2+^ ion can be attributed to the 5-way interaction between the metal and four residues that mediate the shear during domain movement (Fig. 6).

The regression model is a variant of principle component analysis, and its coefficients furnish a detailed histogram of contributions to transition-state stabilization (Fig. 6(b)). The dominant five-way interaction term has a definitive interpretation: at the moment of transition state stabilization, *motions of all four D1 switch side chains and the active-site Mg^2+^ ion are tightly coupled, such that their simultaneous movement can reduce the free energy of the transition state.*^20^ This interpretation implies that the change in side-chain packing that occurs during domain motion occurs essentially simultaneously with some, as yet undocumented, change in the configuration of the Mg^2+^ ion in Mg^2+^·ATP and that this configurational change allows the metal ion to interact more tightly with the transition state. Thus, this multi-mutant cycle is in substantial quantitative agreement with the conclusions drawn from the two-way thermodynamic cycle involving the intact TrpRS enzyme and the Mg^2+^ ion described in the section on Identification of the D1 switch.

### A novel, modular thermodynamic cycle

If the interpretation of high-order coupling described in the section on Combinatorial mutagenesis is correct, then it should also be the case that the two domains that rotate relative to the active site should also be coupled together in the transition state by similar free energies.^44^ We recently confirmed the prediction quantitatively, by constructing and carrying out a modular thermodynamic cycle.^44^ Modular cycles are enabled for TrpRS by the characterization of Urzymes^62–64^ (and potentially of protozymes^26^). As the CP1 and ABD domains are definitively deleted, the TrpRS Urzyme itself plays the role of a molecular knockout. Its remaining catalytic activity thus measures the impact of simultaneously losing both modules. We therefore constructed each modular perturbation necessary to complete the cycle by adding back either CP1 or the ABD to measure the effects of each modular deletion separately (refer to Fig. 1).

**FIG. 7.**
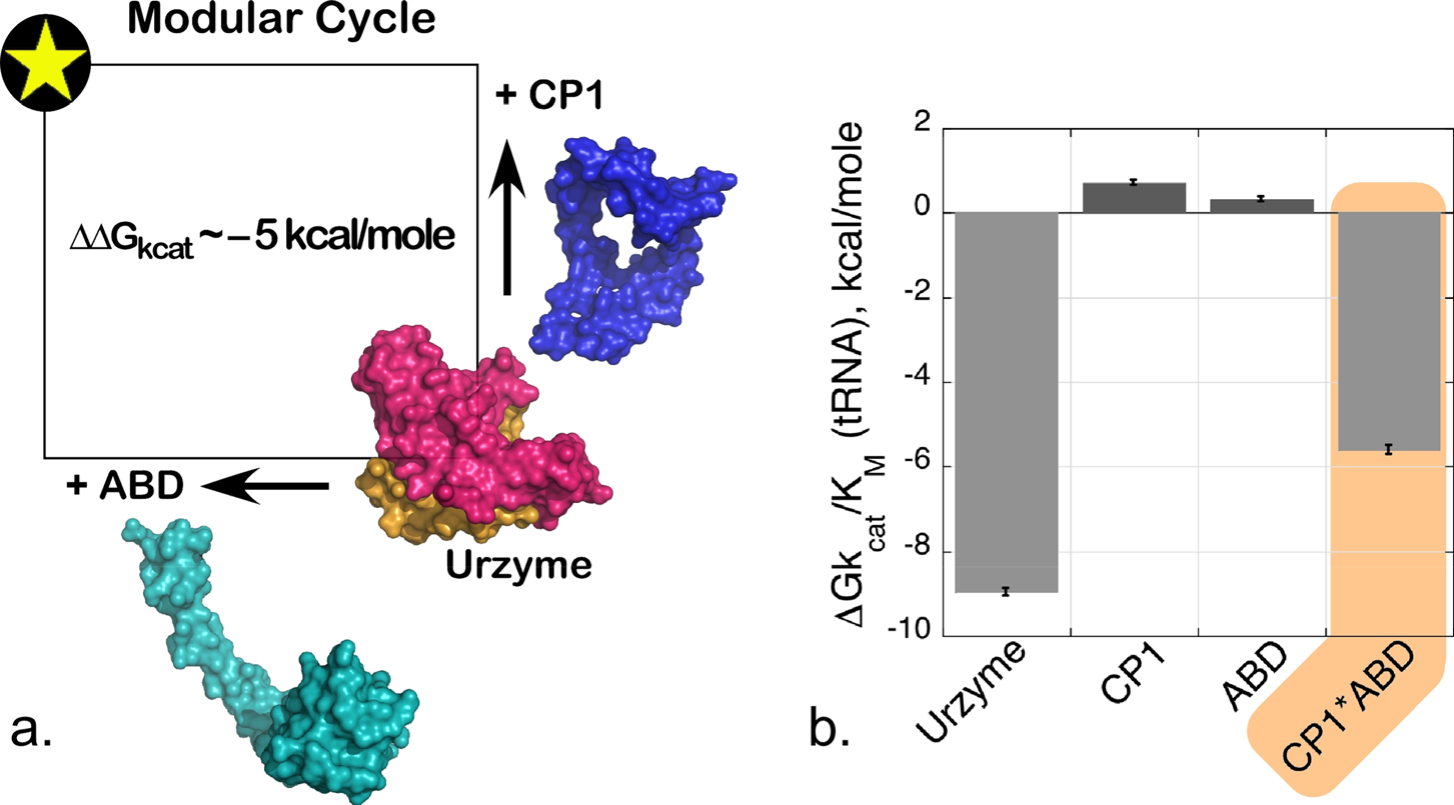
Modular thermodynamic cycle implicating interdomain coupling. (a) Schematic diagram showing the two-way modular variation; adding either the CP1 or ABD domain to the TrpRS Urzyme. (b) Histogram of the free-energy contributions to ΔGk_cat_ for tRNA aminoacylation. Neither domain contributes favorably to catalysis in the absence of the other, and the energetic coupling free energy (shaded amber) is quantitatively the same as that observed for the D1 Switch·Mg^2+^ coupling interaction. R^2 ^= 0.98 for the model, with P(F) < 0.0001. All histograms have Student t-test probabilities <0.001.

Combinatorial domain perturbation (Fig. 7) reveals that neither CP1 nor the ABD contribute materially to transition state stabilization by the Urzyme alone, yet the presence of both modules together in full length TrpRS boosts the catalytic effect by 10^5^-fold! Non-additivity in the resulting modular thermodynamic cycle measured the free energy coupling between the two mobile domains to be −5 ∼kcal/mole in aminoacylation of tRNA^Trp^. This coupling energy closely matched that obtained in the five-way multi-mutant cycle.^20,44^

**FIG. 8.**
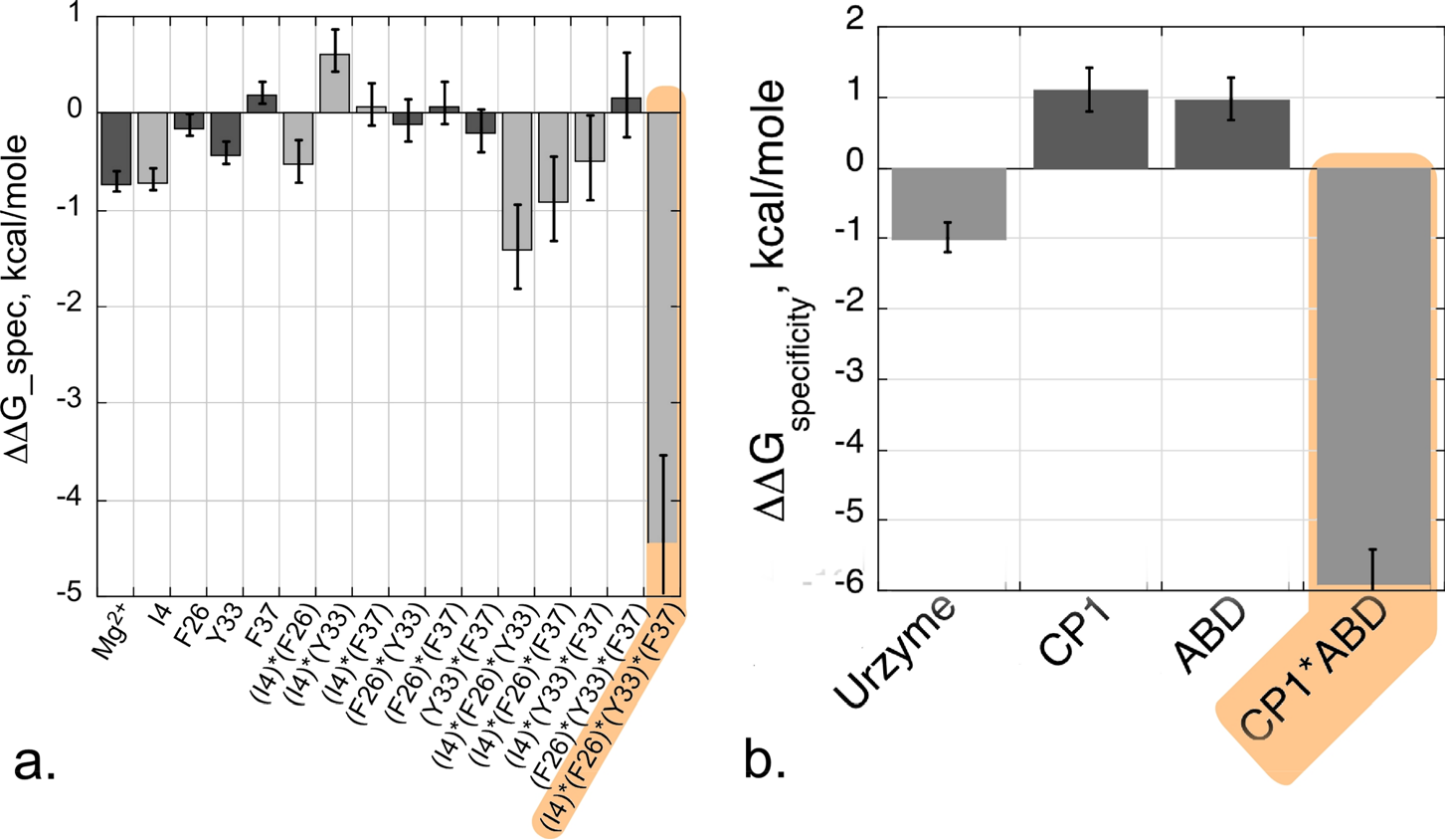
Energetic coupling between D1 side chains and between domains contribute equally to specificity and catalysis. (a) Histogram comparable to that for the combinatorial mutant cycle in Fig. 6(b). (b) Histogram comparable to that for the modular cycle in Fig. 7. In both (a) and (b), the dependent variable is the specificity for tryptophan vs. tyrosine, ΔG(k_cat_/K_M___Trp_)/ΔG(k_cat_/K_M___Tyr_). R^2 ^= 0.99 for the specificity model, with P(F) and all Student t-test probabilities <0.0001.

Multi-mutant and modular models validate one another in specificity as well as in catalysis. The 4-way interaction between I4, F26, Y33, and F37 also makes a similar contribution to the specific recognition of Tryptophan vs. Tyrosine (k_cat_/K_M_)_Trp_/(k_cat_/K_M_)_Tyr_ in tryptophan activation.^30^ Simultaneous motions of CP1 and the ABD are necessary in order to achieve a fully functional TrpRS catalysis and specific tryptophan recognition: in the absence of either domain, the overall rate acceleration falls by ∼10^−5^-fold, almost exactly to that of the Urzyme alone, and the free energy of discriminating between tryptophan and tyrosine falls from ∼6 kcal/mole to 1 kcal/mole (Fig. 8).^30,44^

### TrpRS multi-state behavior is coupled to catalysis and hence functionally relevant

**FIG. 9.**
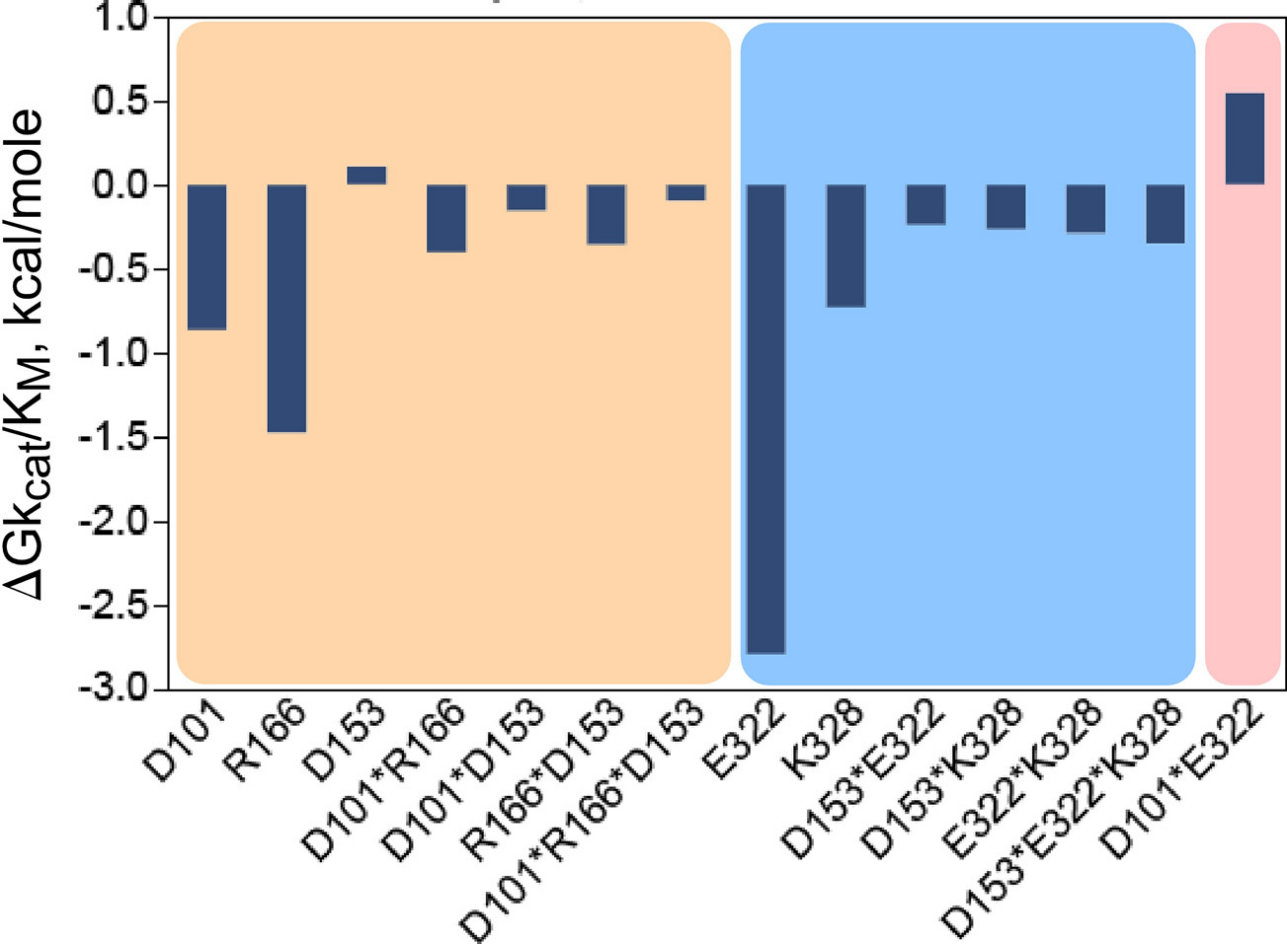
Significant effects from the 5-way combinatorial mutagenesis of active-site residues in alkaline phosphatase.^61^ In contrast to the highly coupled pattern observed in Fig. 6, high-order coupling is not observed, the intrinsic effects are dominant, and connections are limited to local networks denoted by the three shaded backgrounds.

The coupling evident in Fig. 6(b) is notable for several reasons. First, it is contrary to that expected for higher order factorial experiments^65^ in which intrinsic effects normally dominate, and higher order interactions diminish in magnitude. That expected behavior has been verified in a comparable five-dimensional full factorial analysis of the catalytic roles of residues in the active site of alkaline phosphatase^61^ (Fig. 9) whose catalytic side chains link only into small clusters that are themselves dominated by the intrinsic effects of individual side chains. Here (Fig. 6(b)), the dominant effect, by a substantial margin, is the highest order interaction term. Second, the highly cooperative nature of protein tertiary structures suggests the possibility that any sufficiently high-dimensional combinatorial mutagenesis would result in high-order coupling. The pattern in alkaline phosphatase serves as an important “negative control” by showing that high-order interactions do not necessarily result from any high-dimensional mutagenesis experiment. Finally, as discussed by Sunden *et al*., an evolutionary argument holds that high-order coupling is indeed unexpected, and hence represents the product of a complex series of selected properties.^61^ We also have advanced similar arguments regarding the conundrum evident in Fig. 7(b), which suggests that the full-length enzyme could not have evolved by a simple process of accumulating first one, then other of the two domains missing in the Urzyme.^44^

The stunning quantitative agreement between the coupling energies estimated from multi-mutant and modular combinatorial experiments with those anticipated from the characterization of metal-free catalysis^25^ reinforces our earlier suggestions^32,34,38,66^ that domain motions are necessary to form the TrpRS·transition state complex, and hence that differences observed between TrpRS crystal structures are relevant to the catalytic cycle. Thus, the different TrpRS crystal structures probably approximate those states connected by motions necessary for transition-state stabilization. Because the coupling energies contribute to ΔGk_cat_, and hence to transition-state stabilization, they represent the transient interactions that occur only in the transition state. Hence, movement of parts of TrpRS—the D1 switch residues, the CP1 and ABD domains, and the catalytic Mg^2+^ ion—relative to one another is required to generate them, as Fersht noted when interpreting his early directed mutagenesis of the TyrRS active site^50,67–69^ which had to be done without reference to multiple crystal structures.

In view of the recent critique of the functional relevance of domain motions^12^ it is worth discussing these results cautiously. The concluding sections address separate aspects of this question.

### Multi-state behavior is imposed by conformational transition states

**FIG. 10.**
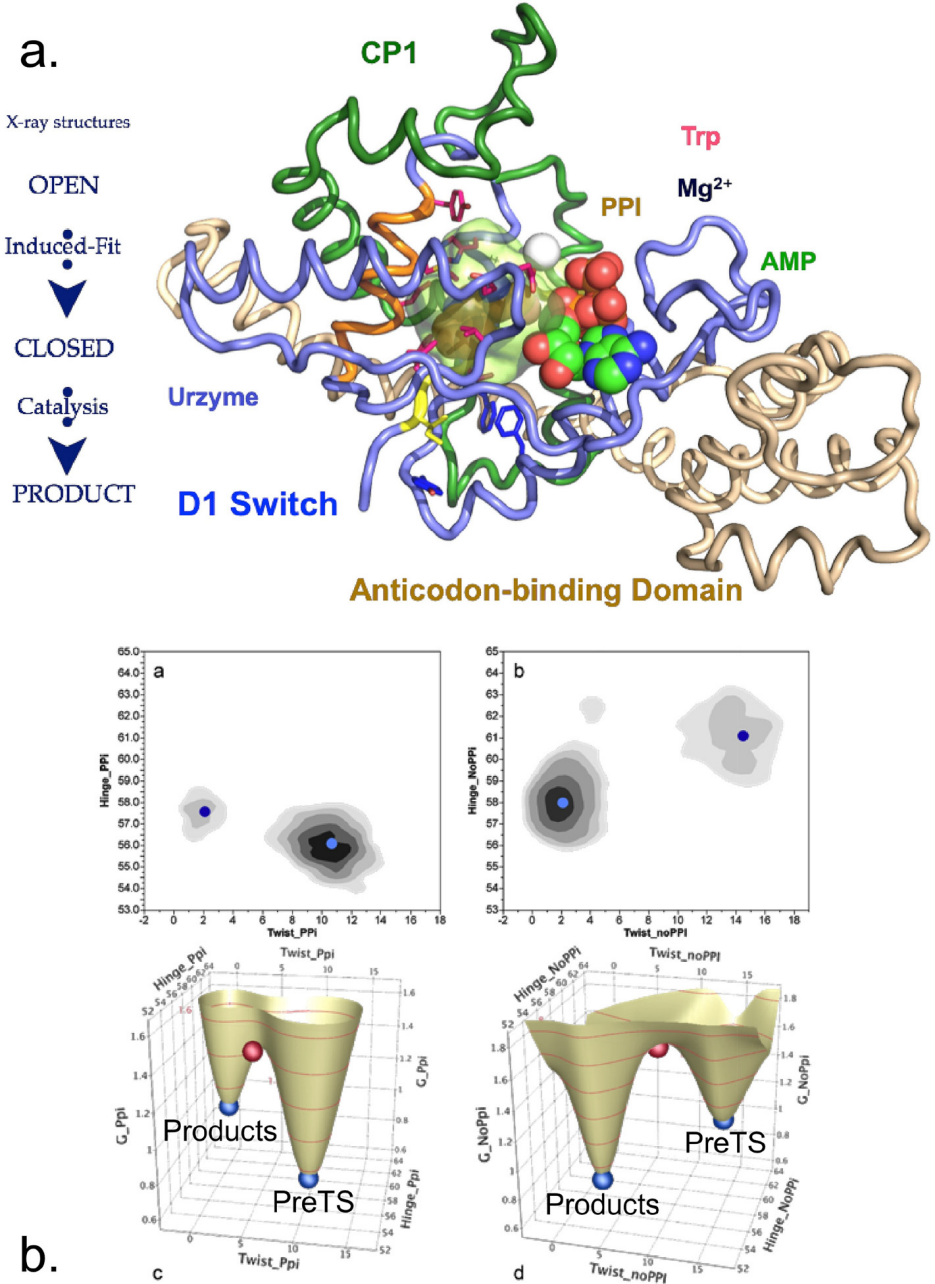
Computational studies relevant to inferences about the relevance of coupling energies.^41^ (a) PATH trajectory [adapted from Weinreb *et al.*, J. Biol. Chem. **289**, 4367–4376 (2014)] implicating the D1 Switch rearrangement in the rate-limiting step in the TrpRS induced-fit conformational change. (b) Free energy surfaces for the catalytic conformational change derived from replica exchange Directed Molecular Dynamics.^71^ Simulation performed with a restraint to retain the bound pyrophosphate (left) show that the PreTS conformation is thermodynamically favored; that performed without restraint shows that pyrophosphate is released, and the Products conformation is favored (right). Reproduced with permission from Chandrasekaran *et al*., Struct. Dyn. **3**, 012101 (2016). Copyright 2016 AIP Publishing. (Multimedia view) [URL: http://dx.doi.org/10.1063/1.4974218.1]10.1063/1.4974218.1.

In order for a protein to exhibit the multistate behavior, its conformation must have multiple distinct potential energy wells, separated by passes of high conformational free energy. We have used a minimum action path algorithm (PATH)^70^ to characterize how the three TrpRS conformational states connect to one another via such conformational transition states (Fig. 10(a) (Multimedia view)).^30,33,41^ Replica exchange discrete molecular dynamics^71^ showed that the conformational transition state for TrpRS catalytic transition is narrowly defined structurally, and that it coincides with a saddle point in the respective conformational free energy landscape^41^ (Fig. 10(b)). Importantly, that landscape refutes the contention^72^ that the saddle point in the free energy surface between multiple states of a protein is relatively flat in the direction normal to the path, and hence helps assure that the most probable path does result from minimizing the Onsager-Machlup action functional expressing the energy dissipation for a stochastic, overdamped system.^73^

In the same study, we also compared the conformational transition states of the TrpRS Induced-Fit transition to those associated with the Pre-power stroke to rigor transition of the myosin VI converter domain^74^ and calcium release by calmodulin. These three transition states are remarkably similar: each entails re-packing of multiple aromatic side chains, which is much slower than other types of repacking. These results, together with our observation that the volume of the tryptophan binding site assumes a minimum just prior to the conformational transition state for the catalytic transition, and hence is likely the moment at which a decision is made regarding whether or not the correct amino acid is bound^30^ all reinforce the conformational transition state as a real, functionally relevant conformation.

### Energetic coupling renders the catalytic NTP hydrolysis contingent on the domain motion

Although TrpRS dynamic behavior appears to be closely linked to catalysis by long-range energetic coupling influences on k_cat_, the domain movement itself probably does not actually lower the energy of the transition state. As noted,^12^ the time frame of the motions is too slow, and the molecular forces generated are too weak to couple directly to transition-state stabilization. Several apparently unrelated observations suggest a novel, perhaps counterintuitive interpretation of the relationship between domain motion and catalysis.

**FIG. 11.**
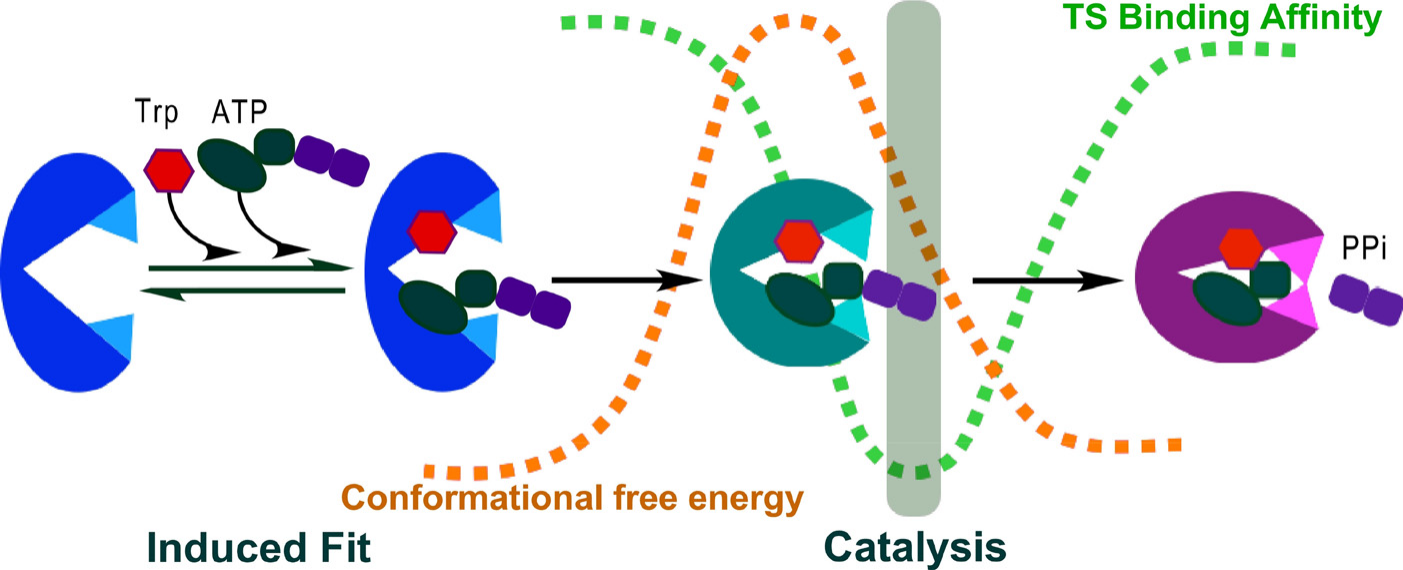
Conformational coupling renders ATP utilization conditional to domain motion. Tryptophan activation by ATP is illustrated schematically, coloring the Open, PreTS, and Products conformations blue, teal, and magenta, respectively. The brown dashed curve reflects transition in conformational free energy; evidence supporting the qualitative aspects of this curve derives from both computational^24,33,40,86^ and experimental^32,34^ studies. The green dashed curve reflects the evolution of transition-state binding affinity, inferred from thermodynamic cycle studies summarized herein.^20,30,44,87,88^ The offset of the two dashed curves means that high transition-state affinity occurs transiently during the domain motion (grey vertical stripe), thereby creating a conditional dependence of transition-state stabilization on domain movement, as is required for efficient free energy coupling. Dashed lines are not intended to indicate the same energy scales, and the time scales are arbitrary.

Rather than contributing directly to transition state stabilization, TrpRS domain motion may contribute instead to dynamic active-site *preorganization*^28^ (Fig. 11). By this hypothesis, motions allow the active site to non-randomly populate configurations complementary to the chemical transition states. The active site is properly configured only transiently, because it coincides with the maximum rate of conformational change and hence exists only for a brief portion of the domain motion. Because preorganization occurs transiently and with a low frequency, the overall chemical reaction may effectively proceed more slowly than it would if the active-site remained pre-organized throughout the substrate residence time.

**FIG. 12.**
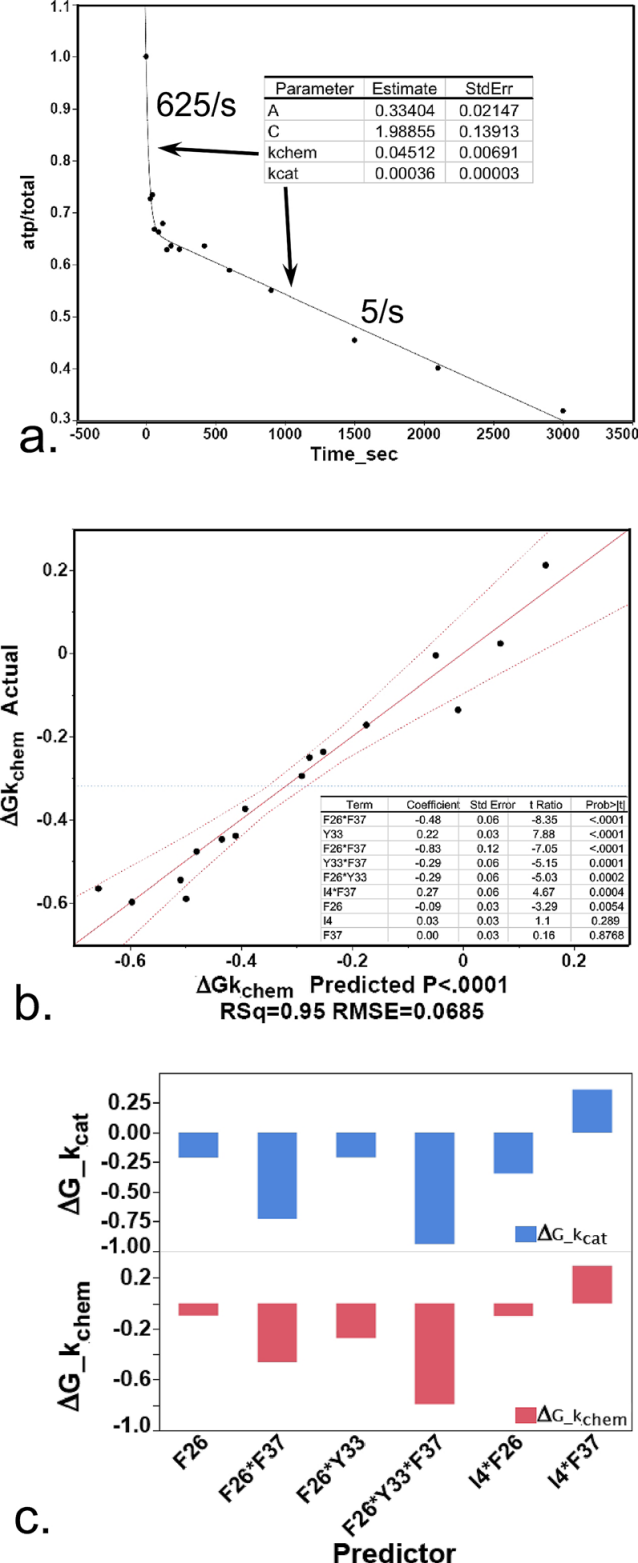
(a) Measurement of the rate of the chemical step of TrpRS-catalyzed tryptophan activation by active-site titration.^60,89^ The fitted line, y = A + exp(−k_chem_*t) − k_cat_*t + C, yields estimates for the first-order rate of the chemical step, k_chem_ and the linear rate corresponding to product release and turnover. The actual turnover number, 5/s, and the ratio k_chem_/k_cat_ furnishes an estimate of the concentration independent rate of the chemical step, which is 625 times faster than the steady-state rate, and is on the order of a few milliseconds. (b) Regression model for ΔGk_chem_ values for WT and the 15 D1 Switch mutant enzymes show that the relevant dependent parameter in pre-steady state active-site titration experiments is the intrinsic rate of the chemical step. The inset table shows estimated energetic contributions of four main effects, i.e., from each D1 site, together with five two-way interactions, in order of decreasing statistical significance. R^2 ^= 0.95 and P(F) < 0.0001. (c) Histograms of regression coefficients for the contributions of each factor for ΔGk_cat_ and ΔGk_chem_.
(ii) The free energy of activation for the chemical step measured in single-turnover active site titration experiments, ΔGk_chem_, is closely correlated to the D1 Switch mutations and their higher-order interactions in almost identical fashion to that observed for the ΔGk_cat_ values (Figs. 12(b) and 12(c)). Turnover itself, estimated from the steady-state rate from such an experiment is, however, uncorrelated with the pattern of mutations. This result is unexpected in light of the evident correlation between mutant sites and ΔGk_cat_ values from steady-state experiments. It can be understood, however, by recognizing that differences in a small fraction of the total reaction time in the latter context are amplified many fold by turnover. The significance of this correlation, evidenced by the very small t-test P-values (P < 0.0001), is that structural perturbations influence primarily the chemical step of the reaction, supporting the conclusion that acceleration of the chemical step itself is tightly coupled to the domain motion.
(iii) Studies of the mechanism by which indolmycin inhibits bacterial TrpRSs^75,76^ show that indolmycin affinity for TrpRS is enhanced by the presence of Mg^2+^·ATP, but not by ATP alone. We have argued elsewhere^75,76^ that this unusual effect can be attributed to a small shift in the crystallographically observed metal position, which moves it from a high-energy (and catalytically competent) position to a low-energy (and catalytically inactive) conformation.

If this hypothesis is correct, then it would account for three observations that previously seemed unrelated:

(i) Active-site titration experiments show that the chemical step occurs in about the same time frame as the domain motions, i.e., milliseconds (Fig. 12(a)). Thus, the chemical reaction itself may actually be controlled by, i.e., retarded, by reduced time during which the active-site retains the catalytically active configuration.

### Coupling catalysis to conformational changes creates vectorial behavior

Enzyme mechanisms, especially those that couple NTP hydrolysis to work and/or information, use sophisticated dynamic networks to transduce the active-site chemistry into domain motions that change the macromolecular binding affinities. The dual energetic coupling of catalysis to domain motion and D1 switch repacking with Mg^2+^ ion movement renders catalytic utilization of ATP conditional, or contingent upon the domain motion. TrpRS domain motions apparently constitute a timing mechanism.

The variety of different TrpRS X-ray structures has identified each conformation with a distinctive spectrum of ligand affinities,^32–34^ including those ligand configurations associated with the transition state. Decelerating the chemical step from what it might be in a more static active site, synchronizing chemical and mechanical events and thereby optimizing the efficiency of free energy transfer. Controlling the pre-organization of the catalytic metal ion can thus be seen as part of an orchestrated sequence that couples these differential binding changes associated with ATP binding, hydrolysis, and product release, to the domain configuration, ensuring that ATP hydrolysis occurs if and only if the conformation changes.^20^

Our recent demonstration that the equilibrium for this catalytic domain movement is driven only by the release of pyrophosphate^41^ appears to be an additional mechanism ensuring that catalysis is coupled to (subsequent) domain movement, presumably regulated by tRNA binding. Using PPi release to change the overall conformational equilibrium resembles the behavior observed for myosin, for which the power stroke is initiated by orthophosphate release, whereas the release of adenosine-diphosphate happens only as the power stroke proceeds toward the rigor state.^77–79^ As both orthophosphate and pyrophosphate are much more strongly solvated by water than is ATP,^80^ an early release of the phosphoryl group may be more widely used to orchestrate the vectorial domain motion in transducing enzymes.

Serial conditionality furnishes qualitatively new insights into the mechanism of chemical free energy transduction and vectorial processes. Perhaps the clearest discussion of these underlying issues is that by Jencks,^13,14^ who outlined how sequential changes in ligand-binding affinity lead to profound changes in the overall free energy of ATP hydrolysis, and recognized the necessity for understanding what he called the “coupling rules.” As the structural landscape of transducing enzymes was as yet unknown, Jencks was mute about what mechanisms actually linked conformational changes to catalysis, and this issue remains unresolved.^81^ Identifying^24,40^ and mutating^29^ key residues associated with the conformational transition state(s), together with modular thermodynamic cycles^44^ to measure energetic coupling between domains, now enhance the understanding of how idiosyncratic structural and mechanistic details implement the coupling rules. These methods should also be useful in studying a large number of other transducing NTPases.

The accompanying paper^82^ describes an improved analysis of TrpRS conformational transition states whose computationally derived thermodynamic and kinetic parameters correlate strongly with structures and kinetic data for all sixteen D1 switch variants, unifying and further validating the results summarized here.

## METHODS

### Identifying potential couples

A full-length enzyme has almost innumerable separate interactions. Consider only 2^N^ experiments obtained by constructing all possible combinations of mutations for one type of substitution per residue in TrpRS (328 residues). This amounts to 2^328 ^= e^1900^ different variants. Clearly, the number of variants must be massively reduced in order even to begin to evaluate long-range energetic coupling in an enzyme. Fortunately, several aspects of domain motion simplify the problem if sufficient structural information is available for the conformational variations along the reaction path.

Many conformational changes consist of rigid-body motions. As these rigid bodies preserve their internal structure, amino acid residues within them can be ignored to a first approximation in designing the combinatorial cycles. Delaunay tessellation discriminates the invariant tertiary interactions from those that change during the structural reaction profile. The remaining, dynamic, interactions constitute a far smaller reservoir of candidates for permutation studies. Rigid body changes therefore entail either hinge-bending or shear, both of which impact small numbers of residues, reducing the number to be considered for combinatorial point mutagenesis.

Algorithms developed for multistate protein design^57–59^ furnish a second, complementary source of useful, residue-specific estimates of free energies associated with the domain motion when crystal structures are available for multiple states. Multi-state design identifies the side chain mutations that alter the relative conformational energies. Dynamic residues identified as potential sites of mutation to alter conformational equilibria form an overlapping, but distinct set of residues.

The Venn diagram of sidechains identified by the Delaunay SNAPP (Simplicial Network Analysis of Protein Packing)^54–56^ and Rosetta energy function tests (Fig. 5) reduces the number of mutants to a manageable number.

Finally, the Rosetta energy function identifies the specific point mutants that have the greatest impact. This contribution cannot be ignored, because it greatly reduces the number of possible mutations at each site to a single amino acid. The process described here thus identifies specific mutations to a sufficiently small number of sites, to compose a sensible set of combinatorial experiments.

### Regression modeling simplifies the data analysis

Regression analysis of experimental results recorded in a factorial design is implemented in most statistics programs. We use JMP statistical discovery software (JMP),^83^ which combines a wide range of state-of-the-art statistical and presentation modules from the SAS system with a very user-friendly interface. Linear models require the use of free energies, which are additive. Thus, all rates and equilibrium constants must be converted first into Gibbs energies. Then, the Gibbs energies contributing either to kcat, K_M_, or k_cat_/K_M_ can be expressed as the linear combination (1)^84^

$$\begin{array}{c} \Delta G_{\text{obs}}=C+\Sigma_{\text{main}}\Delta G_{i}*\left[i\right]+\Sigma_{2-\text{way}}\Delta G_{\text{ij}}*\left[i\right]*\left[j\right]+\Sigma_{3-\text{way}}\Delta G_{\text{ijk}}*\left[i\right]*\left[j\right]*\left[k\right]+\Delta G_{\text{ijkl}}\left[i\right]*\left[j\right]*\left[k\right]*\left[l\right]+\Sigma_{\text{main}}\Delta G_{i\_\_M}*\left[i\right]*\left[M\right]+\Sigma_{2-\text{way}}\Delta_{\text{Gij}\_M}*\left[i\right]*\left[j\right]*\left[M\right]+\Sigma_{3-\text{way}}\Delta_{\text{Gijk}\_M}*\left[i\right]*\left[j\right]*\left[k\right]*\left[M\right]+\Delta G_{\text{ijkl}\_M}\left[i\right]*\left[j\right]*\left[k\right]*\left[l\right]*\left[M\right], \end{array}$$ (1)

where i−l are either 1 or −1 depending on whether the residue at position i is wild-type or mutant, and M is 1 or −1 depending on whether the catalytic metal is Mg^2+^ or Mn^2+^.

A decisive advantage of this approach to the analysis is that coefficients for main effects and interaction terms furnish intrinsic energetic contributions and coupling free energies from the cross terms in units of kcal/mole. A detailed example, clarifying various questions about the method as an alternative to manual calculation of all quantities was described previously^51^ is discussed in the supplementary material for Ref. 84.
